# Use of platelet-rich plasma on in vitro maturation during bovine embryo production

**DOI:** 10.1590/1984-3143-AR2023-0107

**Published:** 2024-03-22

**Authors:** Eduardo Baia de Souza, Diego Dubeibe Marin, Anelise Sarges Ramos, Bruno Porpino Homobono, Priscilla do Carmo de Azevedo Ramos, Vanessa Cunha de Brito, Gabriela Santos da Cruz, Nathalia Nogueira da Costa, Marcela da Silva Cordeiro, Simone do Socorro Damasceno Santos

**Affiliations:** 1 Laboratório de Fertilização in Vitro, Instituto de Ciências Biológicas – ICB, Universidade Federal do Pará – UFPA, Belém, PA, Brasil; 2 Facultad de Medicina Veterinaria y Zootecnia, Universidad de Ciencias Aplicadas y Ambientales – UDCA, Bogotá, Colombia; 3 Laboratório de Biologia Molecular, Universidade Federal Rural da Amazônia – UFRA, Belém, PA, Brasil

**Keywords:** embryo, fetal bovine serum, in vitro maturation, platelet rich plasm

## Abstract

One of the crucial aspects to be considered for successful in vitro production (IVP) of embryos is the composition of the various media used throughout the stages of this reproductive biotechnology. The cell culture media employed should fulfill the metabolic requirements of both gametes during oocyte maturation and sperm development, as well as the embryo during its initial cell divisions. Most IVP protocols incorporate blood serum into the media composition as a source of hormones, proteins, growth factors, and nutrients. Numerous studies have suggested Platelet-Rich Plasma (PRP) as a substitute for fetal sera in cell culture, particularly for stem cells. Therefore, the objective of this study is to assess the potential use of PRP as a replacement for fetal bovine serum (FBS) during oocyte maturation for in vitro production of bovine embryos. During in vitro maturation (IVM), cumulus-oocyte complexes (COCs) were allocated into the following experimental groups: Group G1 (IVM medium with 5% PRP); Group G2 (MIV medium with 5% PRP and 5% SFB); Group G3 (MIV medium with 5% SFB); and Group G4 (MIV medium without either PRP or SFB). Subsequently, the cumulus-oocyte complexes were fertilized with semen from a single bull, and the resulting zygotes were cultured for seven days. Cleavage and blastocyst formation rates were assessed on days 2 and 7 of embryonic development, respectively. The quality of matured COCs was also evaluated by analyzing the gene expression of HSP70, an important protein associated with cellular stress. The results demonstrated that there were no significant differences among the experimental groups in terms of embryo production rates, both in the initial cleavage stages and blastocyst formation (except for the G4 group, which exhibited a lower blastocyst formation rate on D7, as expected). This indicates that PRP could be a cost-effective alternative to SFB in the IVP of embryos.

## Introduction

Despite the extensive research that has already been conducted in this field, in vitro embryo production (IVP) remains the subject of numerous studies aimed at enhancing the technique by refining the conditions to which gametes and embryos are subjected. The goal is to achieve improved results, including a high number of quality embryos, as well as increased transfer and birth rates in the target species (Stroebech et al., 2015). One critical factor for the success of reproductive biotechnology is the quality of the media employed, which must accurately mimic the in vivo physiological microenvironment in both biochemical and physicochemical terms (Abd El-Aziz et al., 2016).

One of the main components of oocyte maturation and embryo culture media is blood serum, with fetal bovine serum (FBS) being the most commonly used. FBS contains high concentrations of several important molecules necessary for proper metabolic functions during preimplantation embryonic development (Del Collado et al., 2015). However, the use of serum presents challenges that can directly impact pregnancy outcomes, including contamination and a lack of standardized results (Paradkar et al., 2019), in addition to the high concentration of lipids. This can result in a range of metabolic alterations, leading to reduced cell quality and low cryotolerance (Dubeibe Marin et al., 2019).

A viable alternative to replacing fetal bovine serum during in vitro embryo production is the use of platelet plasmas. Platelet-rich plasma (PRP), owing to its high concentration of growth factors (Maynard et al., 2007, 2010), is strongly associated with functions such as recruitment, proliferation, and cell differentiation, particularly in tissue regeneration (Tobita et al., 2015). PRP has also been linked to increased embryo production and improved quality during oocyte maturation and in vitro zygote culture, thereby enhancing pregnancy and birth rates (Burnouf et al., 2016).

This type of additive has been employed in the in vitro culture of several cell lines, particularly mesenchymal stem cells, due to its ability to promote high cell proliferation rates, lower senescence rates, and higher cell viability. However, little is known about the effects of platelet lysates in bovine IVP, which may prove to be a more viable alternative to the use of fetal bovine serum (FBS) in this procedure. Therefore, the aim of this study is to evaluate the use of PRP as a substitute for FBS during oocyte maturation in the in vitro generation of bovine embryos.

## Methods

### Experimental design

The oocytes were allocated to the following experimental groups: Group G1(88 COCs) (IVM medium with the addition of 5% PRP), Group G2 (93 COCs) (IVM medium with the addition of 5% PRP and 5% FBS), Group G3 (91 COCs) (IVM medium with the addition of 5% FBS), and Group G4 (88 COCs) (IVM medium without the addition of either PRP or FBS). Four independent replicates were conducted, with each replicate containing cumulus-oocyte complexes (COCs) from all experimental groups. Cleavage rates were assessed on day 2, and blastocyst formation was evaluated on day 7 of embryo development.

### Blood collection and platelet-rich plasma production

The protocol for obtaining PRP was adapted from Marques et al. (2014). To this end, blood was collected from adult cows at the time of slaughter and placed in sterile collection tubes sprayed with heparin. All animals, from which blood was collected for PRP production, were vaccinated, and tested for major diseases prior to slaughter (as per slaughterhouse sanitary requirements). The tubes were then transported to the laboratory for processing on ice. Subsequently, the samples were centrifuged at 400 g for 20 minutes at room temperature to separate the blood components. After centrifugation, the supernatant plasma was divided into two equal-volume fractions: the upper plasma fraction (UPF), representing the uppermost half of the supernatant plasma, and the lower plasma fraction (LPF), which refers to the lower half of the supernatant plasma, closer to the leukocyte layer. The UPF was discarded, while the LPF was retained and transferred to another tube for further centrifugation at 800 g for ten minutes. The upper 75% of the supernatant plasma, known as platelet-poor plasma (PPP), was then discarded, and only the lower 25% fraction (PRP) was retained. The PRP was aliquoted and frozen for future use. A platelet count was conducted using the hematological analyzer CELL-DYN RUBY (ABBOTT), where a platelet count of 37,500 per microliter of plasma was recorded.

### In vitro embryo production

All biological materials used in the work were provided to the in vitro fertilization laboratory of the Federal University of Pará by the local SOCIPE slaughterhouse. The material (blood and ovaries) was collected from already slaughtered animals, therefore there is no need for approval by the university's ethics and research committee.

#### Ovary retrieval

The ovaries were obtained from a local slaughterhouse. Shortly after slaughter, the ovaries were washed in phosphate-buffered saline (PBS) and placed in a saline (0.9% sodium chloride) solution at room temperature. They were then transported to the In Vitro Fertilization Laboratory of the Institute of Biological Sciences of the Federal University of Pará within a maximum of 2 hours for follicular puncture.

#### Recovery and selection of Cumulus-Oophorus Complexes (COCs)

Antral follicles measuring 2 to 8 mm were punctured using 40 x 12 needles and 10-mL syringes, and the collected follicular fluid was transferred to 15-mL tubes. After aspiration, the tubes were centrifuged for 5 seconds to separate the supernatant from the pellet containing the COCs (Cumulus-oophorus complexes). The liquid portion was discarded, and the sediment was transferred to a sterile polystyrene Petri dish with a diameter of 60 mm. The COCs were then screened using a stereomicroscopic loupe. COCs with homogeneous cytoplasm, devoid of vacuoles or darkened granules, and with compact and refreshed cumulus oophorus cells were washed and screened in TALP Hepes supplemented with 10% SFB (v/v) and antibiotics.

#### In vitro maturation

The COCs were washed and incubated in IVM medium based on the experimental groups specified in the design (TCM 199 supplemented with sodium bicarbonate, FSH, LH, pyruvate, antibiotics, heparin, and PRP and/or SFB). The incubation was performed in Petri dishes containing 100 μL drops of medium (10 to 13 COCs per drop) under sterile mineral oil in an incubator with 5% CO2, 20% O2, and 75% N2 under a humid atmosphere at a temperature of 38.5 °C for a duration of 20 hours.

#### In vitro fertilization

Frozen sperm from a single Bos indicus bull was used for in vitro fertilization (IVF). The spermatozoa were separated from cryoprotectants and seminal plasma using a discontinuous Percoll density gradient. The mini straw (250 mL) of sperm was thawed in water at 35 °C for 30 seconds. The semen was then layered over an 800 mL Percoll column (45% and 90% gradients) and centrifuged (600 G) for 7 minutes. The supernatant was discarded, and the pellet was washed by centrifugation in 800 mL TALP medium for 3 minutes to remove Percoll residues. After washing, the sperm concentration was determined using a Neubauer chamber and adjusted to 2x106 SPTZ/mL. Spermatozoa were added to fertilization medium drops (80 mL) (TALP supplemented with heparin, penicillamine, hypotaurine, epinephrine, and BSA) along with COCs (20 to 25 per drop). Oocytes and spermatozoa were incubated together under the same conditions as for IVM for approximately 24 hours.

#### In vitro culture

After IVF, the resulting zygotes underwent successive pipetting to remove any remaining cumulus cells and spermatozoa adhering to the zona pellucida. They were then transferred to culture drops and remained there for seven days. Embryonic development was carried out using a co-culture system, where the embryos were co-cultured with the granulosa cells that remained attached to the plate during IVM. The culture medium used was Synthetic Oviductal Fluid (SOF) supplemented with 10% SFB, 1mM glucose, 10μg/mL insulin, 6mg/mL BSA, 80μg/mL pyruvate, and gentamicin. On the second day after fertilization, the rate of zygotes undergoing the first mitotic divisions was assessed, and on the seventh day, the rate of blastocyst formation and their morphological quality were evaluated, assigning them a score ranging from one to three, with one being the highest quality.

### Gene expression evaluation

#### Sample collection and storage

Groups of 20 matured COCs from the experimental groups were stored in microcentrifuge tubes, with oocytes and granulosa cells stored separately. The cumulus cells were separated from the oocytes using hyaluronidase by successive pipetting. The tubes contained 10 μL of RNAlater® (Applied Biosystems, Foster, CA) and were kept in a freezer at -80 °C until use.

#### RNA extraction and complementary DNA synthesis (cDNA)

RNA extraction from the samples was performed using the PicoPure ™ RNA Isolation Kit (Acturus Bioscience, Mountain View, CA, USA) following the manufacturer's instructions. The isolation process of the kit is based on capturing RNA from the samples into columns with silica membranes for purification. After elution, 10 µL of total RNA were recovered for each sample. Reverse transcription to obtain cDNA was performed using the HighCapacity Reverse Transcription Kit® (Applied Biosystems, Foster City, CA, USA) according to the manufacturer's recommendations. After reverse transcription, 20 µL of total cDNA were recovered for each sample. and the samples were stored in a freezer at -20 °C until use.

#### Amplification of target genes

Gene amplification was carried out using real-time polymerase chain reaction (qrt-PCR) with StepOne Real-Time PCR System software (Applied Biosystems) and the SYBR Green® PCR Master Mix commercial kit (Applied Biosystems). Three replicates were obtained for each experimental group, and each sample was individually amplified in triplicate. In each well, 10 µl of the reaction consisted of 8 µl of SYBR Green® PCR MasterMix (Applied Biosystems), with 0.2 mM of each primer (fowad and reverse), and 2 µl of the cDNA sample diluted at a 1:4 ratio. The thermocycling conditions were 95 °C for 10 min, followed by 45 cycles of 95 °C for 15 s and 60 °C for 1 min. After each PCR run, denaturation curve analysis was performed on each sample to detect oligonucleotide dimers and nonspecific products. To assess the amplification efficiency of each transcript, a standard curve was generated by serial dilution of cDNA. The GAPDH gene was amplified as an endogenous control. Gene expression analysis was performed based on the calculation of:


$${2\mathrm{\Delta \Delta Ct}}^{Efficiency-\;\left(Ct\;Target-Ct\;Endogenous\right)each\;sample-\;(Ct\;Target-Ct\;Endogenous)Calibrator}$$ (1)

The characteristics of the selected genes for analysis are described in Table 1.

**Table 1:** Target genes used for gene expression analyses.

**Gene**	**Sequence (5’ – 3’)**	**Product size (base pairs)**	**GenBank Number**
HSP70	F: GAACGCGCTGGAGTCGTA	202	NM_174550.1
	R: ATGGGGTTACACACCTGCTC		
GAPDH	F: CTCAACGGGAAGCTCACTG	132	XM_006065800.1
	R: CTCTGATGCCTGCTTCACCA		

### Statistical analysis

All statistical analyses were performed using SigmaPlot® software (version 12.0). Analysis of variance (One way ANOVA) was conducted to compare the means, using a significance level of 5% (p < 0.05), and when necessary, the Holm-Sidak post-test was applied to assess significant differences.

## Results

### Embryo production

Analysis of the results from in vitro embryo production revealed no significant differences between the experimental groups in the cleavage rate on the second day of culture. However, a statistically significant difference (p < 0.05) was observed on the seventh day of culture in group G4 (the group without the addition of any type of blood supplement), which exhibited a lower rate of blastocyst formation compared to the other treatments. The mean values along with their respective standard deviations are presented in Table 2.

**Table 2:** In vitro embryo production rates in the different groups containing PRP.

	**N (total)**	**Cleavage (D2)**	**Blastocysts (D7)**	**Blastocysts/cleavages**
G1	88	78,430 ± 4,305	35,180 ± 5,424^a^	44,905 ± 6,941^a^
G2	93	82,749 ± 4,660	40,819 ± 5,691^a^	49,390 ± 6,861^a^
G3	91	77,560 ± 7,176	41,329 ± 8,514^a^	53, 081 ± 8,488^a^
G4	88	74,942 ±13,810	17,354 ± 8,190^b^	23,438 ± 10,399^b^

G1: 5% PRP; G2: 5% PRP + 5% SFB; G3: 5% SFB; G4: 0%. Values shown as mean ± standard deviation.

### Gene expression

**Figure 1:**
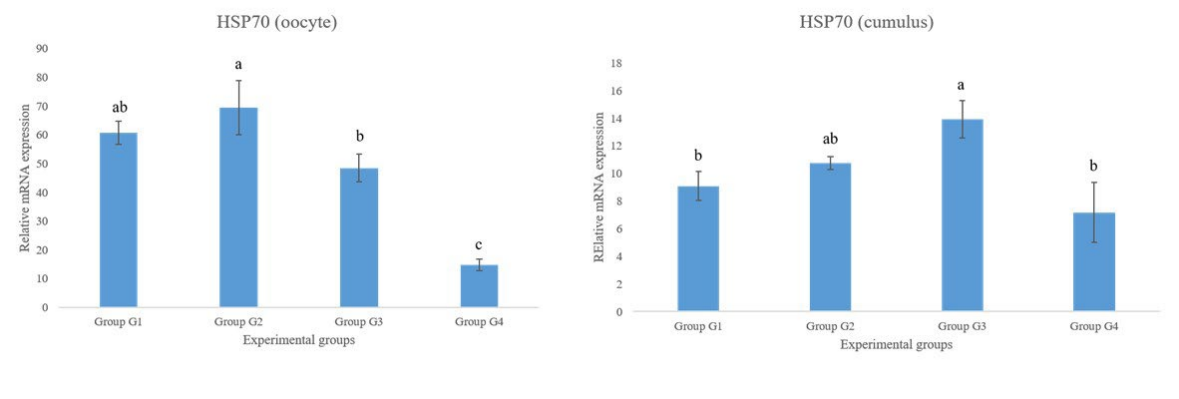
Gene expression of HSP70 in oocytes and granulosa in the different experimental groups. Expression relative to the expression of the endogenous GAPDH gene. Superscripts indicate significant differences (p < 0.05).

Significant differences were observed in the expression of HSP70 in both oocytes and granulosa cells, with the highest relative expression levels observed in the groups receiving blood supplementation (SFB and/or PRP). In oocytes, group G2 (5% PRP and 5% SFB) exhibited higher expression of HSP70 compared to groups G3 (5% PRP) and G4 (0%) (p < 0.001). In granulosa cells, the highest expression was observed in group G3 compared to groups G1 and G4 (Figure 1).

## Discussion

Based on the results obtained, it appears that bovine Platelet-Rich Plasma (PRP) presents itself as a promising alternative. It provides a cost-effective option when compared to the use of Fetal Bovine Serum (FBS) during the 20-hour *in vitro* maturation of bovine oocytes. There were no significant differences in the rates of cleavage and blastocyst formation between treatments (except for group G4 without the addition of a blood component), including the group where both preparations were used together (experimental group G2).

The effect of using platelet derivatives during in vitro embryo production, especially in production animals, is still not well understood. In mice, the inclusion of platelet lysate (PL) or a combination of LP and FBS in the maturation medium led to increased rates of maturation, fertilization, and embryo production compared to the control group with only FBS (Pazoki et al., 2015). However, the impact of supplementing the maturation medium with platelet lysate or other platelet-derived supplements on in vitro production in other species remains unknown.

PRP has been widely used in both in vitro and in vivo models as a source of growth factors due to its composition, which includes transforming growth factor-β (TGF-β), platelet-derived growth factor (PDGF), vascular endothelial growth factor (VEGF), and basic fibroblast growth factor (bFGF) (Sundman et al., 2011). These growth factors play crucial roles in cellular functions essential for embryonic preimplantation development, such as cell proliferation and differentiation (Kawamura et al., 2012). They can also influence oocyte maturation in culture by stimulating granulosa cells and enhancing the rate of oocytes in the second metaphase (M II) (Richani and Gilchrist, 2018).

FBS, as a supplement, also provides growth factors and other important molecules for oocyte maturation and embryonic development, including vitamins, hormones, signaling proteins, and carrier proteins that fulfill the physiological needs of cultured cells in the presence of the blood supplement (Abdel-Wahab et al., 2018). Modifying FBS with PRP does not seem to have a significant effect on increasing or decreasing embryo production rates, as both supplements have complex compositions and provide important molecules for COCs.

Platelets, rich in growth factors, have emerged as one of the most promising alternatives to FBS in culture medium supplementation, particularly in stem cell culture using plasmas and lysates (Astori et al., 2016). Platelet-rich plasma, with its high concentration of growth factors, is closely associated with functions such as recruitment, proliferation, and cell differentiation, particularly in tissue regeneration (Tobita et al., 2015).

The expression of HSP70 was found to be significantly increased in both oocytes and granulosa cells treated with a blood supplement (PRP and/or SFB) compared to the negative control. HSP70 are chaperones involved in various cellular maintenance functions, including the regulation of newly synthesized protein conformation, translocation of polypeptides to organelles such as mitochondria, chloroplasts, and the endoplasmic reticulum (ER), degradation of protein complexes, and regulation of protein activity (Rosenzweig et al., 2019).

The presence of HSP70 during the in vitro maturation of oocytes is associated with increased protection against stress, altering the expression pattern of various crucial genes related to diverse cellular functions essential for the maturation of the cumulus-oocyte complex and subsequent embryonic development. The exogenous addition of HSP70 in the in vitro maturation medium modulates the expression of genes in oocytes involved in the apoptotic pathway (upregulation of BCL2 expression, a gene encoding an anti-apoptotic protein regulating programmed cell death), protection against oxidative damage (increased expression of G6PD, SOD2, GXP1, genes encoding proteins associated with the reduction of reactive oxygen species). Additionally, the presence of HSP70 appears to be linked to microtubules during the stabilization of the meiotic spindle in oocytes, contributing to the gamete maturation process (Stamperna et al., 2021).

HSP expression is associated with the cellular response to stress conditions and is involved in the regulation of apoptosis and elimination of damaged proteins, which are important functions for maintaining cell viability under stressful conditions (da Costa et al., 2019; Shan et al., 2020). The difference in HSP70 expression may be attributed to the stress caused to COCs by the blood supplements in the culture medium. Increased HSP70 expression has previously been observed in bovine oocytes matured in the presence of FBS, which may act as a cellular stress factor, indicating the influence of serum on the overall amount of transcripts in oocytes and cultured embryos (Warzych et al., 2007). PRP showed a similar effect on HSP70 expression; however, the exact mechanism behind the increased number of transcripts for this protein remains unknown.

## Conclusions

The use of Platelet-Rich Plasma (PRP) during the in vitro maturation of bovine Cumulus-Oocyte Complexes (COCs) has shown promise. It ensures a comparable rate of embryo production as when FBS is utilized. The expression of selected oocyte quality marker gene did not exhibit significant changes in the groups using PRP, thereby justifying the exclusion of FBS in the maturation medium. However, due to the variability in PRP production protocols and the dependence of PRP composition on various factors, including preparation methods, further research is necessary to standardize the optimal production protocol for this supplement and achieve more consistent outcomes. Additional studies are also required to assess the effects of PRP on oocyte nuclear maturation in cattle and understand the impact of the supplement on embryonic culture. These studies should examine both the rate of blastocyst formation and the morphological and genetic quality of the resulting embryos.
